# Evaluating the Impact of the Health Navigator Model on Housing Status Among People Experiencing Homelessness in Four European Countries

**DOI:** 10.3390/healthcare13212805

**Published:** 2025-11-04

**Authors:** Juan Esteban Guzman-Benitez, Tobias Fragner, Tamara Alhambra-Borrás, Ascensión Doñate-Martínez, Vicent Blanes-Selva, Juan M. García-Gómez, Simona Barbu, Julia Gawronska, Maria Moudatsou, Ioanna Tabaki, Katerina Belogianni, Pania Karnaki, Miguel Rico Varadé, Rosa Gómez-Trenado, Jaime Barrio-Cortes, Lee Smith, Alejandro Gil-Salmerón, Igor Grabovac

**Affiliations:** 1 Department of Social and Preventive Medicine, Center for Public Health, Medical University of Vienna, Kinderspitalgasse 15/1, 1090 Vienna, Austria; 2 École des Hautes Études en Santé Publique (EHESP), 15 Avenue du Professeur Léon-Bernard, 35043 Rennes, France; 3 School of Medicine and Population Health, Faculty of Health, University of Sheffield, Western Bank, Sheffield S10 2TN, UK; 4 Polibienestar Research Institute, University of Valencia, Calle del Serpis, 29, 46022 València, Spain; 5 Biomedical Data Science Lab—ITACA Institute, Universitat Politecnica de Valencia, Camino de Vera, s/n., 46200 Valéncia, Spain; 6 European Federation of National Organisations Working with the Homeless (FEANTSA), 194 Chaussee de Louvain, 1210 Brussels, Belgium; 7 Centre for Health, Performance and Wellbeing, Anglia Ruskin University, East Rd, Cambridge CB1 1PT, UK; 8 PRAKSIS-Programs of Development, Social Support and Medical Cooperation-Non-Profitable Association, Stournari 57, 104 32 Athens, Greece; 9 Civil Law Non-Profit Organisation of Preventive Environmental and Occupational Medicine PROLEPSIS, Fragkokklisias 5, 151 25 Marousi, Greece; 10 Department of Nutritional Sciences, Faculty of Life Sciences and Medicine, King’s College London, 150 Stamford Street, London SE1 9NH, UK; 11 General Directorate of Social Services, Council of Family, Youth and Social Policy, Community of Madrid, Calle de Serrano, 60, 28006 Madrid, Spain; 12 Foundation for Research and Biosanitary Innovation in Primary Care, Avenida de la Reina Victoria, 21, 28003 Madrid, Spain; 13 HM Faculty of Health Sciences, University Camilo José Cela, Castillo de Alarcón, 49, 28692 Madrid, Spain; 14 HM Hospitals Research Institute, 28692 Madrid, Spain; 15 International Foundation for Integrated Care, Linton Road, Oxford OX2 6UD, UK; 16 Department of Health and Social Work, International University of Valencia, Calle del Pintor Sorolla, 21, 46002 València, Spain; 17 Department of Social Work, Complutense University of Madrid, Avenida Complutense, s/n, 28040 Madrid, Spain

**Keywords:** homelessness, housing, cancer prevention, determinants of health, integrated care

## Abstract

**Background:** People experiencing homelessness (PEH) face significant health disparities and systemic barriers to healthcare, elevating their risk for cancer and other chronic diseases. To tackle PEHs’ challenges in accessing cancer preventive care, the CANCERLESS project implemented the Health Navigator Model (HNM)—a person-centered intervention that utilizes trained Health Navigators to provide tailored support and facilitate service access. Recognizing housing as a key determinant of health, this analysis assessed changes in housing status associated with participation in the HNM among CANCERLESS participants in Austria, Greece, Spain, and the UK. **Methods:** This was a secondary analysis of cross-national data collected during a single-arm interventional study. Of 652 enrolled PEH, 277 (42.5%) completed the HNM intervention follow-up and were included in the analysis. Changes in housing status from baseline to follow-up were categorized using the European Typology of Homelessness and Housing Exclusion (ETHOS) and treated as an ordered outcome. Descriptive statistics were complemented by a cumulative link mixed model with a participant random intercept to estimate the association between time (follow-up vs. baseline) and housing transitions among completers, adjusting for age, residence/legal status, and daily smoking. **Results:** Participants had a mean age of 47.4 (SD 13.8), primarily identified as male (64.1%), reported upper secondary education (33.9%), and were from Western European countries (39.7%), with varying housing situations. Among intervention completers, time (follow-up vs. baseline) was associated with higher odds of being in a higher ETHOS category (OR = 1.49, 95% CI = 1.02–2.20, *p* = 0.042), consistent with a modest improvement in housing status. Larger estimates were observed among migrants without legal documents (OR = 24.13, 95% CI = 6.41–90.89, *p* < 0.001), while daily smoking was associated with lower odds (OR = 0.33, 95% CI = 0.11–0.96, *p* = 0.041); other residence status categories were not statistically significant. **Conclusions:** Suggesting that tailored, navigation-based models, such as the HNM, may be linked to improved housing stability for PEH, these findings can inform piloting and context-aligned integration of the HNM within public health strategies as an alternative approach to address the complex, interconnected health and social needs of PEH. However, the lack of a comparison group and high attrition limit the results’ conclusiveness, and future evaluations should aim to include assessments of housing-associated contextual factors.

## 1. Introduction

Homelessness represents a severe and escalating public health crisis across Europe. The European Federation of National Organizations Working with the Homeless (FEANTSA) estimates that almost 1.3 million individuals in Europe face homelessness on any given night, a figure that underscores the scale of this intricate social issue [1]. Within the European context, people experiencing homelessness (PEH) are not only deprived of stable and adequate housing but are also systematically excluded from essential support services [2], including health and social care [3,4,5,6]. This exclusion results in substantial health disparities, with PEH facing a higher burden of communicable and non-communicable diseases, higher rates of premature mortality, and more frequent acute care utilization and hospitalizations compared to the housed population [7,8,9,10].

Among the most critical health challenges for this community is the elevated risk of cancer, and, thus, cancer-related mortality [11,12,13]. Systemic barriers—such as the absence of sufficient health insurance, difficulties navigating complex and fragmented health and social care systems, competing survival priorities, and experiences of stigmatization and discrimination—severely limit access to cancer screening, early diagnosis, and treatment [11,13,14,15]. Consequently, cancer is often diagnosed at later, more advanced stages with exacerbated symptoms, leading to poorer prognoses and increased mortality rates among PEH.

In response to these challenges, the EU-funded CANCERLESS project (“Cancer prevention and early detection among the homeless population in Europe: Co-adapting and implementing the Health Navigator Model”) was initiated to co-develop, pilot, and evaluate the novel Health Navigator Model (HNM) [16]. Employing participatory research methods, the project brought together PEH as well as professionals from both general and homelessness-specific health and social care settings to tailor the HNM specifically to overcome barriers to cancer prevention services for PEH in Austria, Greece, Spain, and the UK. The HNM is a person-centered approach that uniquely integrates principles of two established frameworks. It draws from the Patient Navigation Model, which provides individualized, practical assistance to help people navigate complex healthcare systems—such as scheduling appointments, arranging transportation, and coordinating services [17]. Simultaneously, it incorporates the Patient Empowerment Model, which focuses on increasing individuals’ autonomy and agency by fostering self-management skills, promoting shared decision-making, and enabling them to become active advocates for their own health and well-being [18]. The resulting co-designed HNM is built on trust and aims not only to provide immediate support but also to equip individuals with the skills and confidence to manage their health needs more effectively in the long term.

As the central pillar of this model, specially trained Health Navigators—who either had a professional background in health or social care or were peer workers—were appointed at each pilot site. Their training focused on increasing knowledge of homelessness and cancer-related risk factors, enhancing communication skills, and developing competence in building trusting relationships with PEH. In practice, these Health Navigators delivered the intervention directly at the pilot sites, conducting primary prevention activities (e.g., one-on-one health needs assessments, guidance on educational workshops, and assistance with health insurance issues) and secondary prevention activities (e.g., arranging cancer screenings and providing accompaniment to medical appointments).

While the primary objective of the HNM within the CANCERLESS project was to improve access to cancer-preventive services, its integrated, person-centered approach aimed to comprehensively address the multifarious needs of PEH. Recognizing that health outcomes are shaped by the conditions and contexts in which people live, the intervention inherently engaged with the social and structural determinants of health in a broader sense [19], therefore, allowing Health Navigation also to yield positive effects on other critical life domains. Housing stability, in particular, is closely linked to cancer-prevention pathways: unstable housing is associated with lower uptake of screening and vaccination, disrupted continuity of primary care and follow-up, and higher prevalence of modifiable risk factors, making it difficult to prioritize health [4,20,21]. Accordingly, we treated housing status as a relevant secondary outcome, recognizing that greater stability may facilitate engagement with preventive services and longer-term health [22,23].

Against this backdrop, the current analysis aimed to assess changes in housing status associated with HNM participation on the housing situations of participating PEH across four diverse European contexts: Austria, Greece, Spain, and the UK. Specifically, changes in participants’ housing status (i.e., housing transitions) following the intervention were analyzed, exploring which demographic and social factors, as well as risk behaviors, may have influenced these outcomes. By examining this secondary, yet vital, impact of the HNM, this research seeks to understand the broader potential of navigation interventions to address the intertwined health and social needs of PEH, contributing needed evidence for the development of more integrated and effective public health strategies for this often-overlooked community.

## 2. Materials and Methods

### 2.1. Study Design

This secondary analysis used data collected during the HNM implementation, which adopted an interventional, non-randomized, single-arm pre-post design conducted across 16 pilot sites in Austria, Greece, Spain, and the UK between June 2022 and December 2023. Data collection employed both quantitative and qualitative methods, tailored to the local contexts of the four countries. The key variables assessed for this analysis included bespoke items and adapted questions designed to capture cancer risk behaviors and modifiable risk factors relevant to the context of PEH.

### 2.2. Participants and Outcomes

Eligible participants were adults (aged 18 or older) who were experiencing homelessness as defined by the European Typology of Homelessness and Housing Exclusion (ETHOS) set out by FEANTSA [24], had no existing or past cancer diagnosis, demonstrated sufficient command of a language spoken at their respective pilot site, and provided written informed consent. Exclusion criteria were a current or previous cancer diagnosis or a cognitive disability that would prevent them from giving informed consent. Health Navigators conducted recruitment at pilot sites, which included community organizations, shelters, outreach programs, day-care centers, and healthcare facilities serving PEH. Data were collected at baseline (i.e., upon entry into the study) and at follow-up (i.e., upon completion of the individual intervention pathway), following standardized protocols determined by the CANCERLESS consortium. Responses were populated into an online data management platform developed for the project [22,23]. For this analysis, the sample was restricted to participants who completed both baseline and follow-up assessments. Item-level missingness was low across variables used in this analysis (≤2.5% for items with any missingness); core variables in the primary model had no or negligible missingness. Baseline characteristics were summarized for all enrolled participants to contextualize attrition.

The primary outcome of interest was the change in housing status, operationalized as transitions across the four conceptual categories of ETHOS, with housing status being modelled as a four-level ordered outcome: roofless, houseless, insecure, and inadequate. We did not use the 13 operational ETHOS categories because small cell counts would have produced unstable estimates and precluded reliable inference. Key demographic and social factors were collected at baseline, including age, residence status (economic migrant, asylum seeker, refugee, undocumented migrant), and daily smoking status (yes/no), to explore their associations with housing transitions from baseline to follow-up. At follow-up, participants reported satisfaction with navigator contact and perceived support (process/implementation measures); these were collected post-exposure and were not intended as baseline covariates. Covariates were prespecified for theoretical relevance and parsimony. Age was included as a basic demographic factor within social determinants frameworks relevant to PEH [19,25,26]. Residence status offered clarity in the difference between nationals and immigrant categories and documentation status, which aligns with eligibility for benefits and access to services that may influence housing status transitions [2,4]. Daily smoking was included as an indicator of modifiable health risk with known implications in prevention and access to health and social services in this priority population [13,27]. Given the sample size and ordered categorical outcome, a parsimonious set was used to reduce overfitting and multicollinearity.

### 2.3. Statistical Analysis

The analysis was conducted in several stages. First, descriptive statistics were calculated for the ETHOS categories, as well as relevant covariates, at both baseline and follow-up.

Second, to assess changes in housing status associated with the HNM’s implementation, a paired Wilcoxon signed-rank test was applied to ETHOS scores, with confidence intervals and pseudomedian estimates reported. The ETHOS score is derived from categorizing participants’ housing status according to the ETHOS criteria, with higher scores indicating greater housing stability [24]. Thus, a positive change in the score reflects a transition to a more stable housing situation. This non-parametric test was chosen to detect the overall direction and significance of change in the paired data.

Third, to further quantify the association with time and investigate which demographic and social factors were associated with housing transitions, a cumulative link mixed model (CLMM) was fitted. This statistical model is suitable for ordinal outcome data (i.e., the ETHOS categories) and accounts for the paired nature of the data, incorporating participant ID as a random effect to control for within-subject correlation. The model was built sequentially, first including only the time point (baseline versus follow-up) as a fixed effect to estimate the unadjusted time association. Subsequently, baseline covariates (age, residence status, and daily smoking) were added as fixed effects to create an adjusted model. Model fit was evaluated using the Akaike information criterion (AIC) and log-likelihood values. The proportional odds assumption was assessed by comparing the results with a nominal effects model and through graphical diagnostics, with no violations observed. Odds ratios and 95% confidence intervals were calculated for all predictors. We considered alternative coding of ETHOS, including a binary ‘more stable’ versus ‘less stable’ grouping and reduced-level schemes. However, given sparse transitions and the sample size, these models were unstable and either failed to converge or degraded model fit; we retained the four-level ordinal specification, which satisfied model assumptions. Process measures collected at follow-up were qualitative variables assessing respondents’ satisfaction. They were not included in adjusted models due to temporal ordering (post-exposure).

Finally, the robustness of findings was assessed through several approaches. A complete-case analysis (listwise deletion) was conducted for inferential models, with all main predictors fully observed and outcome missingness among completers negligible. For categorical items with very low missingness, simple mode imputation was applied in descriptive summaries and sensitivity checks; the primary CLMM estimates were unchanged. Alternative covariate specifications were explored, including gender, education, and citizenship (whether participants were born in the EU or not); these models either failed to converge or did not improve the fit (AIC increased from 1232.60 to 1235.45), supporting the robustness of the primary results. Attrition bias was evaluated by comparing baseline characteristics between PEH who completed and those who did not complete the intervention. These additional analyses provided context for the observed associations and informed the generalizability of findings.

Visualization of predicted probabilities versus observed ETHOS categories was performed, with most points clustering close to the higher categories, indicating sufficient agreement between the model’s predicted and observed outcomes. All analyses were performed using R statistical software (version 2024.12.1+563), employing the “ordinal” package for CLMM [28]. No imputation was used in the primary analyses; multiple imputation using the “mice” package was explored for sensitivity analyses that included additional covariates with sporadic missingness [29], yielding conclusions consistent with the complete-case results.

## 3. Results

A total of 652 participants were enrolled in the study, of whom 277 (42.5%) completed both baseline and follow-up assessments (“completers”), while 375 (57.5%) were lost to follow-up (“dropouts”).

The mean age at baseline was 47.4 (SD 13.8). Men comprised the majority in both groups, while around one-third were women. The largest share of participants held lower or upper secondary education, with similar proportions across both groups (54.2% and 60.8%). Participants represented a diverse range of backgrounds, with the majority being citizens of Austria, Greece, Spain, and the UK (405; 62.9%). Economic migrants comprised a substantial proportion of the sample, including asylum seekers (108; 16.8%), refugees (11; 1.7%), and those without legal documents (120; 18.6%).

The sample exhibited high levels of housing instability, with several participants categorized as facing rooflessness or houselessness at baseline (40.2% and 27.1%, respectively). Participants showed elevated health risk behaviors, including a high prevalence of daily smoking (64.3%), with varying frequencies of alcohol (56.8%) and psychoactive substance use (23.5%), and varying engagement in health-promoting behaviors such as physical activity (moderate: 58.9%; vigorous: 68.7%) and handwashing (>2 times a day: 81.4%). Comparisons at baseline between those who completed the intervention and those who did not showed that dropouts were more likely to have lower educational levels, less stable housing, and higher rates of daily smoking. Completers had a more even distribution across education and housing categories, and slightly higher rates of health-promoting behaviors, such as regular hand washing. No significant differences in other health risk behaviors were observed between groups. In terms of region of birth, the majority of completers and dropouts were UK and Western European citizens. Further details on sample characteristics are shown in Table 1, with additional information reported in Appendix A Table A1.

Comparing baseline and follow-up scores among completers using the Wilcoxon signed-rank test revealed a statistically significant shift in ETHOS scores over time (V = 1304, 95% CI = 0.00008–1.000, *p* = 0.035), with an estimated pseudomedian change of 0.50. The mean ETHOS score increased from 2.19 at baseline to 2.30 at follow-up, with a mean paired difference of 0.10. Medians were unchanged (equal to 2 at both time points), and the median paired difference was 0, indicating that while the central tendency remained stable, there was a small but statistically significant positive shift, consistent with a modest positive change in housing situations among participants.

**Figure 1:**
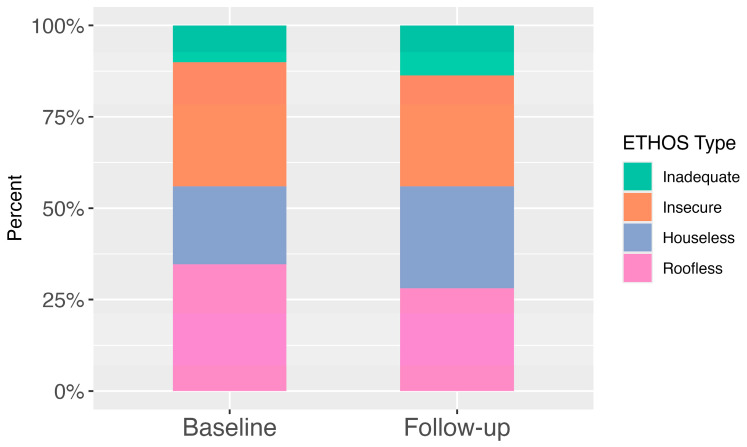
Overall proportion of ETHOS categories among those completing the intervention. Note: Higher ETHOS categories reflect greater housing stability (Roofless < Houseless < Insecure < Inadequate).

**Figure 2:**
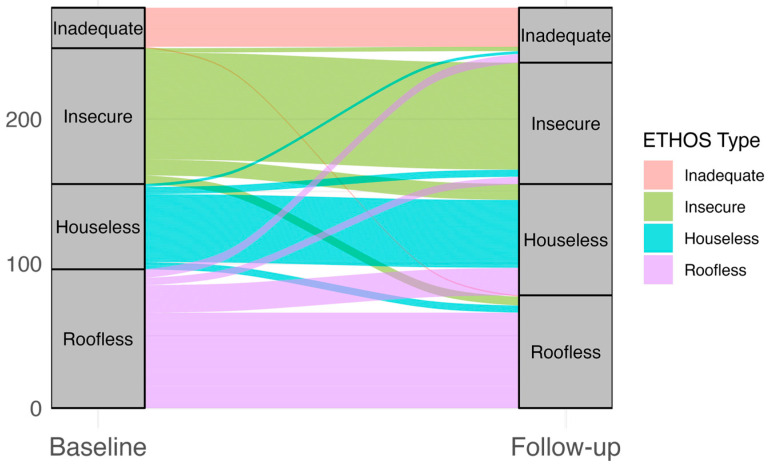
Individual transitions in ETHOS categories. Note: Higher ETHOS categories reflect greater housing stability (Roofless < Houseless < Insecure < Inadequate).

Figure 1 shows the overall proportion of participants in each ETHOS category at both time points among completers, showing a slight shift toward less severe categories at follow-up (higher categories represent less severe forms of homelessness). Figure 2 depicts individual transitions between ETHOS categories from baseline to follow-up, highlighting the movement of participants across housing situations. At baseline, among completers, the proportions were as follows: Inadequate (10.1%), Insecure (33.9%), Houseless (21.3%), and Roofless (34.7%). At follow-up, the proportions were: Inadequate (13.7%), Insecure (30.3%), Houseless (27.8%), and Roofless (28.2%).

In the adjusted CLMM among completers, time (follow-up vs. baseline) was associated with higher odds of being in a higher ETHOS category (OR = 1.49, 95% CI = 1.02–2.20, *p* = 0.042), consistent with a modest improvement in housing status at follow-up. Age at baseline was associated with a slight, non-statistically significant increase in the odds of being in a higher ETHOS category (OR = 1.04, 95% CI = 1.00–1.08, *p* = 0.065). Lack of residence status as an economic migrant was strongly associated with higher odds of being in a higher ETHOS category (OR = 24.13, 95% CI = 6.41–90.89, *p* < 0.001). Daily smoking was associated with lower odds of being in a higher ETHOS category (OR = 0.33, 95% CI = 0.11–0.96, *p* = 0.041). Other residence status categories were not statistically significant (see Table 2).

Model fit was good (AIC = 1232.60, log-likelihood = −606.30). The variance of the random intercept was 14.79 (SD = 3.8), which quantifies the degree to which individual participants differed in their underlying housing status. In this context, the higher variance corresponds to substantial individual heterogeneity among the participants. Threshold coefficients for the ordinal categories were as follows: Roofless/Houseless = −0.40, Houseless/Insecure = 2.52, and Insecure/Inadequate = 7.44. These coefficients represent cut-off points between adjacent categories and are essential to interpret the probability of transitioning between levels of housing status.

The proportional odds assumption was evaluated by comparing the CLMM to a nominal effects model. The nominal model did not improve fit (AIC and log-likelihood were identical), supporting the proportional odds assumption for these data. This indicates that the estimated time association is consistent across all ordinal housing category thresholds, allowing for straightforward interpretation of the odds ratios and supporting the use of the CLMM for this analysis.

**Figure 3:**
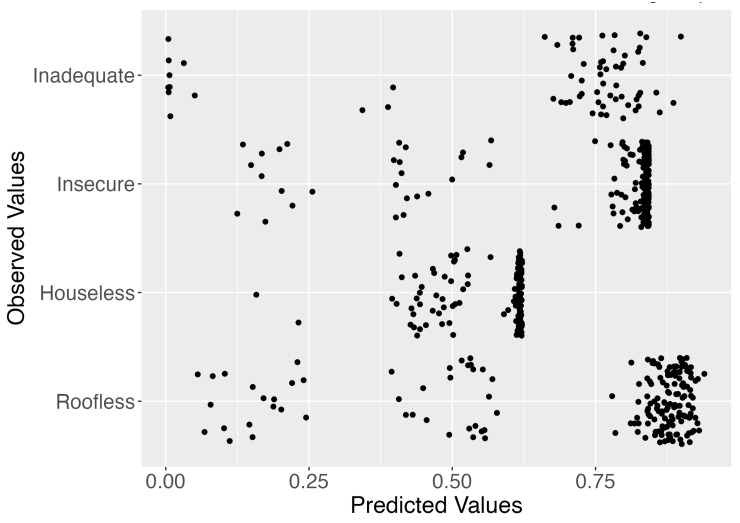
Predicted vs. observed values for ETHOS subgroups. Note: Predicted probabilities for each ETHOS subgroup (x-axis) plotted against observed ETHOS categories (y-axis) for individual participants. Points that cluster at higher predicted values within their observed category indicate better model performance, while wider dispersion reflects misclassification or uncertainty.

The predicted versus observed plot for the CLMM (see Figure 3) demonstrates good model discrimination, with most points clustering close to the higher categories. This indicates strong agreement between predicted probabilities and observed outcomes, particularly for the “Roofless” and “Inadequate” categories. Some dispersion was seen in the intermediate categories (“Insecure” and “Houseless”), suggesting slightly lower predictive accuracy in these groups. Overall, the model provides reasonable discrimination and validity for classifying across ETHOS categories.

## 4. Discussion

The findings of this secondary analysis provide insights into the potential of the HNM in supporting housing stability and housing situations of PEH across diverse European contexts. Implementing the HNM in Austria, Greece, Spain, and the UK was associated with a statistically significant positive shift in housing status, consistent with the notion that navigation interventions with a person-centered and integrated approach may address not only access to health services but also critical social and structural determinants of health. This is a notable finding, given that housing was considered a secondary outcome to the CANCERLESS project’s primary focus on health, and, in particular, cancer prevention. The person-centeredness itself may partially explain this pattern, as it works to rebuild trust with priority populations who have experienced institutional failure or trauma [30]; by fostering relationships based on empathy, respect, and choice, the HNM may increase engagement with services [16]—a potential step toward housing stability for PEH. At the same time, in our sample, transition generally showed shifts toward less severe housing categories, which in turn may facilitate engagement with preventive health services and continuity of care [21,25,31].

Additionally, some other covariates, such as being an economic migrant without legal documents, were strongly associated with higher odds of being in a higher ETHOS category, while no statistically significant associations were found for the other residence status categories (asylum seekers and refugees). This may be the case since the HNM included low-barrier access, not requiring proof of legal status among participants, thereby welcoming undocumented individuals, a group often excluded from mainstream programs. On the other hand, daily smoking was significantly negatively associated with housing status, which may reflect higher underlying psychosocial stress and related health-risk behaviors, shown to be associated with reduced utilization of health interventions and less engagement with preventive healthcare services [27].

Our findings align with previous research, which has reported that Patient Navigation models, designed to overcome healthcare system barriers and enhance access to care services, are linked to increased service uptake and social outcomes, such as housing and employment stability [32,33]. Thus, by using an integrated approach, the HNM may help address both health and social needs, with associations consistent with reduced disparities and progress toward equity in these domains [26,34]. Few studies have directly measured changes in housing status as an outcome of navigation programs [35], making our analysis a contribution to the literature on the wider effects of navigation approaches, particularly in cancer (preventive) care for PEH.

The results of this analysis support the need for integrated care interventions that simultaneously address health and social needs for PEH, a population for whom adverse outcomes in one domain can interact with and exacerbate those in the other [2,25]. Moreover, the larger associations in housing status among undocumented migrants participating in this study suggest that the HNM may be particularly beneficial when coupled with social and legal support services. Such an integrated approach may facilitate access to healthcare and may offer pathways to more stable housing and other key determinants of health for priority populations [6]. Policymakers may consider piloting and, where appropriate, scaling integrated navigation programs, such as the HNM, as part of comprehensive strategies to help reduce health and social inequities in marginalized communities, contingent on further comparative evidence. Practically, adding navigation components to routine services, considering local capacity and legal contexts, and implementing standardized process measures with stronger retention monitoring would generate the evidence necessary for larger comparative studies. Ongoing refinement of both intervention models and evaluation strategies will be vital for progressing public health efforts in this underserved community.

### Strengths and Limitations

The integrated design of the HNM intervention, along with its application across various regions and contexts, is a key strength of this research, offering valuable evidence for innovative and inclusive public health strategies. Advanced statistical modelling, using a CLMM, supported analysis with the ordinal scale, capturing the direction of change. Since the intervals between ETHOS categories are not uniform, the estimated odds reflect shifts towards less severe exclusion rather than equal unit changes. The use of CLMM was, therefore, appropriate despite significant attrition and missing data, which are common in studies involving highly mobile populations such as PEH. This has been demonstrated in other large cohort and systematic studies focusing on PEH, which have reported similar challenges in maintaining follow-up and data completeness [36,37].

However, the current analysis has some limitations and areas for improvement. The notable rate of attrition, which led to a “completers-only” analytical strategy, raises concerns about selection bias and restricts the generalizability of the findings. This is particularly important as individuals who dropped out may have faced more severe living conditions, meaning their absence from the follow-up analysis could underestimate the full challenges experienced by those most marginalized and, in turn, further restrict generalizability. There are some notable differences between individuals who completed the intervention and those who left it prematurely, which can bias the observed associations, raising the possibility of attrition bias. For example, people who completed the intervention had a more stable housing status at baseline, which could have contributed to their adherence. Additionally, factors such as social stigma, discrimination, and access to informal support networks that may not have been captured in these data may also influence health outcomes and engagement with prevention services.

Furthermore, the use of a single-arm, non-randomized design limits causal inference without a comparison group, as this design makes it challenging to determine whether observed changes are due to the intervention itself rather than external factors, such as time-varying confounding, regression to the mean, or concurrent services [38]. External validity is also restricted because findings might not apply beyond the participating sites and service ecosystems, nor to PEH who did not stay engaged in the follow-up. We considered including the pilot country in adjusted models; however, sparse data and non-convergence prevented stable estimation. The absence of standardized process measures, such as navigator contact frequency and linkage outcomes, complicates the interpretation of mechanisms and hampers the assessment of dose–response relationships. We performed sensitivity checks, including baseline comparisons of completers and dropouts and testing alternative covariate specifications. For a small number of categorical items with very low missingness, simple mode imputation was used in descriptive and sensitivity analyses; the conclusions remained consistent with the primary complete-case results. Additional methods, such as inverse probability weighting or tipping point analyses, could be considered in future work if the data allows.

Despite these methodological challenges, the analysis offers valuable insights into the potential of tailored interventions to support housing stability among PEH, with observed associations indicating modest improvements, while emphasizing the need for more rigorous and inclusive research designs in future work.

## 5. Conclusions

This analysis demonstrates how the HNM—an integrated, person-centered care approach co-designed with PEH and care professionals—may be linked to improvements in housing situations and housing stability among PEH across various European contexts. The findings suggest that integrated care interventions, which combine joint healthcare with attention to social and structural determinants of health, are feasible and may support movement toward less severe forms of housing exclusion. This transition may, in turn, facilitate engagement with preventive healthcare services, continuity of care, and overall well-being; however, further research is required to test these pathways.

While the study’s limitations, including attrition, selection bias, and the absence of a control group, highlight the challenges of conducting research with PEH, the observed associations—especially among participants without legal documents at baseline who received support in obtaining documentation—underscore the importance of integrating legal and social support into navigation models. Ultimately, these findings support consideration of piloting and, where appropriate, adapting the HNM as part of broader national public health strategies to help reduce health and social inequities for PEH, contingent on further comparative and process evaluations. Future research should prioritize more rigorous evaluation designs and focus on adherence to better assess associations and mechanisms. This work can open the door to more inclusive and innovative navigation models that advance equity and health outcomes in PEH as well as other priority populations across diverse contexts worldwide.

## Figures and Tables

**Table 1:** Sample characteristics at baseline and follow-up.

Characteristic	Completers Baseline	Dropouts Baseline	Completers Follow-up
**Sociodemographics**			
*N*	277	375	277
Gender: Male (%)	61.7	65.9	
Gender: Female (%)	37.5	33.6	
Age Mean (*SD*)	49.4 (13.3)	45.9 (14.0)	--
Age Median (*IQR*)	50 (20)	47 (19)	--
EU Citizen (Western Europe/UK) (%)	63.9	56.3	--
**Education Level**			
Lower Secondary (%)	27.1	28.5	
Upper Secondary (%)	27.1	32.3	
**Health Risk Patterns**			
Daily Smoker (%)	59.2	68.0	57.0
Alcohol Use: Never (%)	47.3	40.3	50.5
Alcohol Use: Monthly or less (%)	20.6	16.0	18.0
Psychoactive Substance Use: Never (%)	80.1	73.9	81.2
Meal Frequency: Two/day (%)	40.4	30.1	45.8
Meal Frequency: Three/day (%)	35.0	44.3	29.6
Condom Use: Rarely or Never (%)	31.4	32.8	33.2
Moderate Physical Activity, i.e. 10–30 min/day (%)	52.0	64.0	59.2
Vigorous Physical Activity, i.e. 10–30 min/day (%)	57.8	76.8	70.8
Handwashing: 2–4 times/day (%)	53.4	50.7	49.1
Handwashing: 5+ times/day (%)	34.3	26.1	35.0
Sun Exposure: Sometimes (%)	31.0	37.3	31.8
Sun Exposure: Often (%)	30.3	27.5	27.4
**ETHOS Subgroup Categories**			
Roofless *n* (%)	96 (34.7)	166 (44.3)	78 (28.2)
Houseless *n* (%)	59 (21.3)	118 (31.5)	77 (27.8)
Insecure *n* (%)	93 (33.6)	73 (19.5)	84 (30.3)
Inadequate *n* (%)	28 (10.1)	16 (4.3)	38 (13.7)
NA *n* (%)	1 (0.4)	2 (0.5)	--

**Table 2:** CLMM results regarding ETHOS categories. Note: Odds ratios greater than 1 indicate higher odds of being in a more stable category at follow-up versus baseline. Thresholds are estimated from the data.

Predictor	Odds Ratio	95% CI (Lower)	95% CI (Upper)	*p*-Value	Interpretation
Time post (intervention)	1.49	1.02	2.20	0.0418	Significant positive time association (follow-up vs. baseline)
Age at baseline	1.04	1.00	1.08	0.0649	Not significant; slightly higher odds
Residence status at baseline (Asylum seeker)	0.40	0.08	2.11	0.2801	Not significant; possible lower odds
Residence status at baseline (Refugee)	0.46	0.01	26.64	0.7062	Not significant; very wide 95% CI
Residence status at baseline (Economic migrant in an irregular situation)	24.13	6.41	90.89	2.53 × 10^−6^	Strong, significant positive association
Daily smoking status	0.33	0.11	0.96	0.0412	Significant negative association

## Data Availability

The data presented in this study are available upon reasonable request from the corresponding author due to ethical reasons.

## Abbreviations

The following abbreviations are used in this manuscript:

AIC	Akaike information criterion
CLMM	Cumulative link mixed model
ETHOS	European Typology of Homelessness and Housing Exclusion
HNM	Health Navigator Model
PEH	People experiencing homelessness

## Appendix A

**Table A1:** Baseline characteristics of the study sample (completers and dropouts).

Variable/Category	Completers (*n* = 277)	Dropouts (*n* = 375)
**ETHOS Category (T0)**		
Roofless	96	166
Houseless	59	118
Insecure	93	73
Inadequate	28	16
NA	1	2
**Education Level**		
Early childhood	18	11
Primary	40	55
Lower secondary	75	107
Upper secondary	75	121
Post-secondary non-tertiary	22	23
Short-cycle tertiary	10	9
Bachelor’s degree	29	27
Master’s degree	1	4
Doctoral degree	0	0
**Gender**		
Male	171	247
Female	104	126
Non-binary	0	1
Other	1	1
Prefer not to say	1	0
**Smoking Status**		
Non-daily smoker	113	119
Daily Smoker	164	255
**Alcohol Use**		
Never	131	151
Monthly or less	57	60
2–4 times/month	46	63
2–3 times/week	23	46
4+ times/week	20	46
**Psychoactive Substance Use**		
Never	222	277
Monthly or less	9	21
2–4 times/month	11	18
2–3 times/week	8	15
4+ times/week	24	33
**Meal Frequency**		
One	48	50
Two	112	113
Three	97	166
Four or more	19	41
None	1	0
**Condom Use**		
Always	56	56
Most of the time	39	25
About half the time	20	21
Sometimes (<50%)	16	23
Rarely or never	87	123
**Moderate Physical Activity**		
10–30 min/day	144	240
30–60 min/day	22	60
1–2 h/day	29	33
2–3 h/day	4	6
3+ h/day	6	6
**Vigorous Physical Activity**		
10–30 min/day	160	288
30–60 min/day	10	31
1–2 h/day	10	15
2–3 h/day	2	0
3+ h/day	2	2
**Handwashing**		
Never	6	6
Once	28	77
2–4 times	148	190
5 or more	95	98
**Sun Exposure**		
Never	22	20
Rarely	82	106
Sometimes	86	140
Often	84	103
**Birth Region**		
Asia	8	3
Eastern Europe	42	53
Latin America	7	23
Middle East/Central Asia	10	8
Missing	1	2
North Africa	3	27
Other	11	25
Southeast Asia	8	0
Sub-Saharan Africa	8	23
UK	52	77
USA	2	0
Western Europe	125	134
